# „Alexander Kraft 2019: Berliner Blau. Vom frühneuzeitlichen Pigment zum modernen Hightech-Material“ und „Bettina Bock von Wülfingen (Hg.) 2019: Science in Color. Visualizing Achromatic Knowledge“

**DOI:** 10.1007/s00048-021-00311-w

**Published:** 2021-09-07

**Authors:** André Karliczek

**Affiliations:** Thüringer Universitäts- und Landesbibliothek Jena, Jena, Deutschland

**Alexander Kraft 2019: *Berliner Blau. Vom frühneuzeitlichen Pigment zum modernen Hightech-Material.*** Berlin: GNT-Verlag, geb., 312 S., 90 überwiegend farbige Abb., 39,80 EUR, ISBN: 978-3-86225-118‑6.

**Bettina Bock von Wülfingen (Hg.) 2019: *Science in Color. Visualizing Achromatic Knowledge.*** Berlin: De Gruyter, brosch., 239 S., 100 farb. Abb., 36,70 EUR, 978-3-11060-468‑9.

Farben faszinieren die Menschen nicht nur in ästhetischer Hinsicht oder aufgrund ihrer Verwendungsmöglichkeiten in Kunst, Handwerk und Wissenschaft, sondern sie sind als Differenzwerte unserer visuellen Wahrnehmung, als erfahrungsgebundene und -gewordene Empfindungen wesentlich daran beteiligt, wie wir die Welt in einer für uns funktionellen Weise sehen und verstehen können. Gleichwohl wohnt Farben eine gewisse Unschärfe inne, entziehen sie sich doch seit jeher gleichermaßen fachwissenschaftlichen wie kulturellen Bestimmungsversuchen. Entsprechend sind Auswahl und Qualität der jährlich erscheinenden Bücher zum Thema Farbe überreich und lassen sich kaum mehr in Gänze überblicken. Die beiden hier zu besprechenden Werke zeigen dies eindrücklich.

Das Berliner Blau ist eine erstaunliche Farbe. Das tiefblaue Blutlaugensalz darf als eines der ersten künstlich gewonnenen Pigmente überhaupt gelten und entfaltete nach seiner Entdeckung zu Beginn des 18. Jahrhunderts eine ganz außergewöhnliche Wirkmächtigkeit. Diese Geschichte verfolgt Alexander Kraft in seinem detailreich geschriebenen Buch in sieben Kapiteln, die von der zufälligen Entdeckung über die wirtschaftliche Verwertung und globale Verbreitung bis hin zu seiner heutigen Funktion als modernes Hightech-Material reichen. Kraft zeigt dabei anschaulich, wie sich das blaue Pigment in neuen Kontexten immer wieder neu erfand und macht die Farbe damit nicht nur zum Thema, sondern zum zentralen Akteur des Buches. Neben der Verwendung von Berliner Blau in der Malerei und Textilfärberei sind vor allem seine Vielfältigkeit und kontinuierliche Nutzung bis heute besonders erstaunlich und in dieser Breite bislang noch nicht erzählt. So war und wird es unter anderem als Analyseinstrument für den Nachweis von Eisen, als sogenanntes Waschblau zur Kompensierung der Gelbfärbung weißer Wäsche oder für ein frühes fotografisches Verfahren, die Cyanotypie, verwendet. Durch leichte Abwandlung des Herstellungsprozesses können zudem ein gelbes und ein rotes Pigment abgeleitet werden, die wiederum neue Eigenschaften aufweisen und weitere Anwendungsmöglichkeiten eröffnen.

Der weitaus größte Teil der Erzählung handelt von den vier bei der Entdeckung involvierten Hauptakteuren – einem Theologen, einem Offizier, einem Alchemisten und einem Apothekergesellen –, deren unterschiedliche Lebenswege nicht nur bis zu ihrem Zusammentreffen in Berlin, sondern jeweils konsequent bis zu ihrem Tode beschrieben werden. Dabei schießt Kraft zum Teil über das Ziel hinaus und referiert zahlreiche Nebensächlichkeiten sehr ausschweifend und detailliert. So finden sich seitenlange Beschreibungen der politischen oder auch gesellschaftlichen Situation, die wenig bis gar keinen Bezug zum Berliner Blau haben. Das Buch verliert den thematischen Fokus oft stark und verirrt sich in historischen Details, auf die ein abrupter Schwenk zurück zum eigentlichen Thema folgt, was den Leser manches Mal irritiert und fragend zurücklässt. Vielfach ist zu merken, dass es dem Autor schwerfällt, einen konzisen Erzählstrang zu entwickeln und aus der zweifellos großen Fülle des recherchierten und zusammengetragenen Quellenmaterials eine spezifische Farbgeschichte zu destillieren. Auf der anderen Seite fehlt es dem Buch an manchen Stellen an farbhistorischer Breite und Aktualität sowie wissenschaftshistorischem Hintergrund. Letzteres wird zum Beispiel dann deutlich, wenn die Akteure des 17. und 18. Jahrhunderts in „seriöse Chemiker oder allgemein Naturwissenschaftler“ sowie in alchemistische „Betrüger“ und „Scharlatane“ eingeteilt werden.

Hierzu passt, dass Kraft nicht nur auf entsprechend aktuelle chemiehistorische, sondern auch auf die reichlich vorhandene farbhistorische Literatur verzichtet, weshalb es der Ausarbeitung oft am notwendigen Forschungsstand fehlt. So hätte dem Buch unter vielem anderen eine Einordnung in den von Alexander Engel in seinem Werk *Farben der Globalisierung* ausgebreiteten Kontext der globalen Bedeutung der Farbmärkte oder ein Blick in die fast schon klassische *Kulturgeschichte der Farbe* von John Gage sehr gutgetan. Auch vermisst der Leser die Erwähnung der Verwendung von Berliner Blau unter anderem beim Cyanometer, bei dem es erstmals zum Messen sogenannter opaker Dünste zum Einsatz kam. Somit erscheint Alexander Krafts *Berliner Blau* als isolierte, gleichwohl lesenswerte Einzelgeschichte, der trotz ihrer Materialfülle wichtige Zusammenhänge entgehen und deren Nutzen für die Leserinnen und Leser mehr in den zusammengetragenen einzelnen Quellen als in der Darstellung einer guten Gesamtgeschichte zu suchen ist.

Eine ganz andere Geschichte oder besser Analyse der Farben schreibt Bettina Bock von Wülfingen in ihrem Sammelband *Science in Color. Visualizing Achromatic Knowledge*. Der im Exzellenzcluster Bild Wissen Gestaltung entstandene Band nimmt eine sowohl ästhetische als auch epistemische Spurensuche der Bedeutung von Farbe und ihrer Verwendung in (natur-)wissenschaftlicher/medizinischer Forschung und Lehre in den Blick. Ganz zweifellos besteht hier ein großes Desiderat an systematischen Arbeiten, da Farben in diesen Bereichen allgegenwärtig sind und viele verschiedene Funktionen übernehmen, der konkrete Umgang mit ihnen aber oft in eingeübten Wissenschafts- und Abbildungspraktiken zu einer Tradition verstetigt ist, die kaum mehr kritisch reflektiert wird.

Der Sammelband enthält dreizehn thematisch breit gefächerte Beiträge und ist in zwei große Teile gegliedert, die – so die Herausgeberin (12) – die beiden Bedeutungsebenen von Farbe repräsentieren. Die ersten sechs Beiträge stehen unter der Überschrift „Color and its meaning for the sciences“, womit die (Nicht‑)Bedeutung von Farben in Bezug auf ihre Nutzbarmachung gemeint ist. Die sieben Artikel des zweiten Teils – „Meaningful colors in the sciences“ – befassen sich dann mit der symbolhaften Bedeutung von Farbe und ihrer Verwendung als Vehikel für achromatische Inhalte. Diese Struktur erscheint auf den ersten Blick griffig, trennt sie doch zwischen dem Bedeutung-Finden über und dem Bedeutung-Setzen durch Farbe. In letzterem Fall überführt die Farbe dabei bestimmte kulturelle Codes in einen neuen Kontext.

Wie wird dies nun konkret eingelöst? Im ersten Teil finden sich unter anderem Beiträge zum diagnostischen Wert verschiedener Falschfarbendarstellungen in der Medizin, Untersuchungen zur Kontinuität kultureller Farbpräferenzen (Chromophobie versus Polychromie) bis hin zur Frage nach einer eigenen Farblogik und Verwendung von Farbe zur Visualisierung vierdimensionaler Objekte. Zwei Beiträge seien exemplarisch kurz dargestellt. Aldo Badano untersucht in „Color in Medical Images“ auf Basis einer empirischen Studie den Einfluss von Farbigkeit in bildgebenden Verfahren wie der Computer- oder Magnetresonanztomografie auf die diagnostische Performanz und über diese auf Therapie und Prognose. Dabei stößt die neuerliche Farbigkeit medizinischer Visualisierungen auf eine monochromatische Tradition der Darstellung mitsamt ihren etablierten Deutungspraktiken. Im Ergebnis zeigt Badano, dass die Nützlichkeit verschiedener Falschfarbendarstellungen (Greyscale, Hot-Iron und Rainbow) von ihrer Aussagekraft in Bezug auf die medizinischen Fragestellungen abhängt. Der Vorteil der Farbigkeit in Bezug auf die bessere Darstellbarkeit qualitativer oder quantitativer Unterschiede wird dabei jedoch begleitet von einem generellen Glaubwürdigkeitsdilemma, das durch die Unkenntnis der Bildproduktion in der klassischen Beziehung zwischen Urbild (Objekt), Medium (CT, MRT etc.), Abbild (Display und Farbschema) und einer jeweils zeitgenössisch und kulturell bedingten Wahrnehmungspräferenz begründet liegt. Im Zentrum von Alexander Nagels Kapitel „Research on Color Matters: Towards a Modern Archeology of Ancient Polychromies“ steht die Bedeutung historischer Polychromien und deren Rekonstruktionen für unser jeweils aktuelles Weltbild. Nagel verweist dabei auf ein vierfaches Problem im 19. Jahrhundert – nämlich einerseits die spärlichen Reste von Farbspuren in standardisierter Form zu dokumentieren, diese in einem zweiten Schritt zu analysieren und drittens authentisch zu rekonstruieren. Schließlich kommt die zeitgenössische Akzeptanz der Rekonstruktion als viertes Problem hinzu.

Im zweiten Teil des Sammelbandes, den „Meaningful colors in the sciences“, finden sich unter anderem Beiträge zur therapeutischen Bedeutung von Grün, zu verschiedenen Farbschemata und deren Nutzen in der Datenanalyse, zur Verwendung von Farbe in geografischen Karten sowie zu Farbtraditionen in komplexen Prozessdiagrammen. Auch hier seien beispielhaft zwei Beiträge kurz vorgestellt. Daniel Baum diskutiert in „An Evaluation of Color Maps for Visual Data Exploration“ insgesamt acht verschiedene Falschfarbgradienten (Color Maps) zur Visualisierung kontinuierlicher Skalarwerte. Dabei geht es im Kern um die Frage, welche Anforderungen ein Farbschema zur Visualisierung erfüllen muss und wo die jeweiligen Stärken und Grenzen liegen. Die vergleichende Darstellung der Farbschemata in Bezug auf unterschiedliche Datensets führt eindrücklich vor Augen, wie stark der visuelle Eindruck und damit der durch die konkreten Farbskalen implizierte Aussagewert jeweils variieren kann.

In „The Use of Color in Geographic Maps“ von Jana Moser und Philipp Meyer geht es um den intentionellen Einsatz von Farbe zur Visualisierung verschiedener Inhalte geografischer Karten. Farbe ist hier kein bloßes Gestaltungsmittel, sondern bietet die Möglichkeit, Zusammenhänge, Assoziationen, Emotionen und so weiter auf Karteninhalte zu übertragen und damit sublim Informationen zu vermitteln. Neben der generellen Entwicklung entsprechender Farbschemata und -standards sucht der Beitrag über einen interkulturellen Vergleich konkrete Farbzuordnungen sowie -traditionen für Nationen auf politischen Karten in Schulatlanten und Weltkarten und den damit verbundenen Weltbildern herauszuarbeiten.

Die Breite der Beiträge ist beeindruckend und erlaubt einen guten Einblick gleichermaßen in historische wie aktuelle Aspekte der Farbforschung. Gleichzeitig sind sie aber thematisch und methodisch derart disparat, dass sich kein Gesamtbild zu ergeben vermag. Die angestrebte Zweiteilung des Bandes wirkt aufgesetzt und wird inhaltlich nicht eingelöst. Bedurft hätte es eines konstitutiven Metatextes, der erst einmal an konkreten Beispielen die strukturgebenden Bedeutungsschichten entwickelt oder aber diese aus den Fallstudien deduziert. Die Schwierigkeit, trennscharfe Kategorien zu bilden, verweist dann auch auf den zweiten blinden Fleck der Anthologie, nämlich eine eigene Definition von Farbe und ihres Gebrauchs in den Wissenschaften aufzustellen, an der sich die einzelnen Untersuchungen hätten ausrichten können. Dem eingangs skizzierten Desiderat einer analytischen Bearbeitung der Bedeutung von Farbe in der Wissenschaft hilft der Sammelband insofern nicht ab, er erweitert jedoch den farbwissenschaftlichen Horizont.

